# Efficacy of Non-Enhanced Brain Computed Tomography in Patients Presenting to the Emergency Department with Headache after COVID-19 Vaccination

**DOI:** 10.3390/jcm12165279

**Published:** 2023-08-14

**Authors:** Yongtack Lee, Kyuseok Kim, So-Hyun Paek, Hyunglan Chang

**Affiliations:** Department of Emergency Medicine, CHA Bundang Medical Center, CHA University School of Medicine, Seongnam 13496, Republic of Korea; hyinuyu@gmail.com (Y.L.); dreinstein70@gmail.com (K.K.); hyun21400@chamc.co.kr (S.-H.P.)

**Keywords:** brain CT, COVID-19, COVID-19 vaccination, headache, vaccination side effect

## Abstract

Headaches are a common side effect of vaccination against the severe acute respiratory syndrome, coronavirus 2; however, it is usually not necessary to seek emergency medical attention or undergo brain imaging such as non-enhanced brain computed tomography (CT) for routine evaluation of vaccine-related headaches. This study aimed to demonstrate that brain CT is of no clinical benefit to patients presenting to the emergency department (ED) with post-coronavirus disease 2019 (COVID-19) vaccination headaches. This retrospective, single-center observational study used electronic medical record (EMR) data of patients who received the COVID-19 vaccination during the first year of the vaccination program. In total, 914 patients were analyzed, of whom 435 underwent CT (CT group, n = 435; no CT group, n = 475). More female patients visited the ED, and there was no significant sex difference between the CT and no-CT groups. The type of vaccine affected the clinical decision to perform brain CT, but the number of doses did not. The CT rate was relatively high for patients who had received the ChAdOx1 nCoV-19 (Oxford–AstraZeneca) and Johnson and Johnson Janssen (Jansen) vaccines (*p* = 0.004). Focal neurological deficits were present in all cases of abnormalities on non-enhanced brain CT in patients complaining of headaches. Two out of the 435 patients had abnormal brain CT findings (glioblastoma and Rathke’s pouch cyst) at 35 and 32 days after vaccination, respectively. Non-enhanced brain CT should be performed cautiously in patients visiting the ED for post-vaccination headaches only.

## 1. Introduction

Coronavirus disease 2019 (COVID-19) caused by the severe acute respiratory syndrome coronavirus 2 (SARS-CoV-2) has shown significant morbidity and mortality, with >600 million COVID-19 confirmed cases and >6 million deaths worldwide [1]. In South Korea, there have been over 30 million confirmed cases and over 30,000 deaths since the first case reported on 20 January 2020 [2,3]. With the onset of the COVID-19 pandemic, rapid research on various vaccines has been conducted, and vaccines with various mechanisms have been developed [4,5]. Starting with the first inoculation dose administered on 26 February 2021, South Korea is currently administering the fourth-to-fifth dose of COVID-19 vaccine inoculation. With the start of the COVID-19 vaccination program, people were reassured by vaccine development, but fears about the safety of vaccines led to low vaccination rates in the early days. The vaccination rate gradually increased as the policy of social distancing was mitigated only for vaccinated individuals. The safety of the vaccine has been proven in several studies, and the current national second or higher vaccination rate has reached 86.7% [6].

Vaccine side effects are common even if the safety of a given vaccine has been previously proven [7]. In South Korea, the ChAdOx1 nCoV-19 (AZD1222) vaccine manufactured by Oxford–AstraZeneca (AZ) and the Pfizer-BioNTech BNT162b2 COVID-19 vaccine manufactured by Pfizer (Pfizer) are the main vaccines used. The number of patients visiting the emergency department (ED) due to side effects has increased owing to the high COVID-19 vaccination rate. Several side effects, such as injection site pain, musculoskeletal pain, headache, fever, chills, and fatigue, have been reported to arise after vaccination [8,9,10]. We have also learned that vaccine-induced immune thrombocytopenia and thrombosis (VITT) occurs after a COVID-19 vaccination, especially with the AZD1222 vaccine [11,12]. A headache is an important symptom of venous thrombosis, and a delayed headache after adenovirus vector-based COVID-19 vaccination (AstraZeneca, Johnson & Johnson, Sputnik V, and others) is a red flag sign of VITT. In a study conducted by García et al., the criterion for delayed HA was 7 days or later [13].

Headaches are the fourth most common cause of ED visits in the United States, accounting for 2–3% of all visits annually [14]. Among non-traumatic headache patients, a secondary headache occurs in 1 in 25 patients admitted to the emergency room, including 1.4% of the patients with ICH or stroke and 0.5% with CNS infection [14]. According to the International Classification of Headache Disorders, headaches can be categorized into primary and secondary disorders, with primary disorders being much more common. Primary headaches can be treated conservatively in the ED with a focus on prevention after discharge, while secondary headaches are usually emergencies and can be life-threatening if not diagnosed quickly [15], with the risk of permanent neurological sequelae; therefore, imaging studies are essential for differential diagnosis. CT is widely used in medicine. A three-dimensional model through understanding human anatomy is developed and used for reconstructions, and biomimetic scaffolds are developed with micro 3D CT [16,17]. Among these, non-enhanced brain CT is one of the most useful diagnostic tools in the ED. This technique proves instrumental in the accurate identification of medical conditions such as subarachnoid hemorrhage, various cerebrovascular disturbances, and a predominant subset of acute, life-threatening headaches that are categorized as benign primary headaches [18,19,20].

This study shows that non-enhanced brain CT is not clinically helpful for patients who visit the ED with a headache or other symptoms accompanying a headache after receiving a COVID-19 vaccine.

## 2. Materials and Methods

### 2.1. Study Setting

This retrospective single-center observational study utilized an electronic medical record (EMR) database of patients who visited ED for post-COVID-19 vaccination headaches between 26 February 2021, and 26 February 2022. The study facility was a secondary academic hospital in South Korea, with an annual ED admission rate of 70,000 patients/year. This retrospective study was approved by the Institutional Review Board of the CHA Bundang Medical Center, CHA University School of Medicine (2022-04-032-002). Written informed consent was not required because no identifiable patient data was included in the study.

### 2.2. Study Population

All adult patients aged 18 or older who were admitted for headaches after vaccination during the study period were included. When the COVID-19 vaccination was first started in Korea, the target age was recommended to be 18 years or older, and even after that, children were not vaccinated well for the first year. This institution is a hospital where pediatric emergency rooms are separated, and only adult patients over the age of 18 visited during the study period. Patients with underlying diseases that may have headaches due to cerebral hemorrhage, cerebral infarction, cerebral aneurysm, and chronic headaches were excluded. Patients with previous records of brain tumors or brain metastases were similarly excluded from the study. Patients with cerebral hemorrhage, cerebral infarction, cerebral aneurysm, chronic headaches, and history of brain tumors or brain metastases were excluded. A total of 931 patients with post-COVID-19 vaccination headaches visited the ED during the study period, and 914 were included after applying the exclusion criteria.

### 2.3. Data Collection and Data Analysis

Data were collected by reviewing the EMRs. Demographic data (age and sex), clinical information (date and time of arrival at the ED, chief complaint, and discharge), vaccine information (time since injection, vaccine type, and dose schedule), and imaging study status were collected.

Continuous variables were presented as means with standard deviations or 95% confidence intervals, and categorical variables, as frequencies and percentages (%). Chi-square and Fisher’s exact tests were used to compare categorical data. Student’s *t*-test was used to compare continuous variables. Multiple logistic regression analysis was used to analyze the factors associated with CT conduction. Statistical significance was set at *p* < 0.05. IBM SPSS Statistics ver. 22.0 (IBM Corp., Armonk, NY, USA) was used for all statistical analyses.

## 3. Results

Out of a total of 914 patients, 276 patients reported a headache as their chief complaint and brain CT was performed in 246 of 276 patients (*p* = 0.000); there were 30 patients who underwent both CT and MRI. Mean ages of the CT and no-CT groups were 45.74 and 42.98 years, with a significant difference in the mean age, age distribution between the two groups (*p* = 0.010). Pfizer had the highest inoculation rate, followed by AstraZeneca, Moderna, and Janssen. In the brain CT group, 110 patients (25.3%) were vaccinated with AZ, and 30 patients (6.9%) were vaccinated with Jansen, compared to 87 (18.2%) and 15 (3.1%) in the group that did not undergo brain CT. There was significant difference of type of vaccines. (*p* = 0.004).

Among a total of 507 patients (55.5%) who presented to the hospital with a headache < 8 days after vaccination, 223 patients (51.3%) underwent a brain CT. For headaches lasting < 8 days, the number of patients who did not undergo CT was significantly higher. The numeral rating scale (NRS) of the headache was recorded for all except two patients; the average score of the CT group was 2.89, and that of the no-CT group was 1.44, indicating that the average pain intensity of the CT group was higher (*p* = 0.000). Whether analgesics were taken before visiting the ED did not affect performing brain CT. Thirty (6.90%) patients underwent brain MRI after brain CT, which was significantly higher than the number of patients who underwent MRI without brain CT. Patients who underwent brain MRI rejected brain CT during the data review. Most patients were discharged, and the numbers of patients who were hospitalized and discharged against medical advice were higher in the no-CT group (*p* = 0.001) (Table 1).

Table 2 shows four cases showing abnormal CT findings. The 61-year-old patient in the first case in Table 2 was diagnosed with bilateral internal carotid artery (ICA) dissection after outpatient brain CT angiography, so CT and MRI were not performed in the ED. The patient developed bilateral ICA dissection during the Valsalva maneuver, and both rheumatoid and genetic disease tests showed normal findings. In the two patients with cerebral infarction and CT abnormalities, the main symptom was extremity weakness. The other two patients were diagnosed with brain tumors and congenital anomalies.

## 4. Discussion

The novel COVID-19 vaccines have various adverse effects. Headaches were reported as the third most common side effect after vaccination [21], with significant neurologic side effects such as Guillain–Barre Syndrome (GBS) and cerebral venous thrombosis (CVT) occurring in <1 per 1,000,000 doses. The most commonly reported adverse neurological events were headaches, fatigue, dizziness, and syncope [22]. Non-enhanced brain CT was the most commonly used imaging modality for patients visiting the ED for headaches as well as for patients who complained of headaches after vaccination. However, the CTs of patients with non-traumatic headaches were controversial. If non-traumatic headaches are isolated symptoms without local neurological deficits, most patients can safely manage them without radiological imaging. However, most emergency physicians are often under pressure to avoid missing a single diagnosis [23]. This study examined the limited clinical benefits of non-enhanced brain CT for patients who visit the ED with headaches and other symptoms after COVID-19 vaccination.

During the study period, non-enhanced brain CT was performed in approximately 48% of the patients who visited the ED with headaches after a COVID-19 vaccination. Four patients had abnormal CT findings, and two had significant neurological symptoms suggestive of prominent cerebrovascular disease, such as side weakness. MRI confirmed cerebral infarction in two. The other two had glioblastoma and Rathke’s pouch cyst, but no acute disease.

Of the 276 patients who presented with a headache as their chief complaint, 246 underwent brain CT in the ED and 30 did not, with 8 in the former group and 1 in the latter group being hospitalized. One patient without prominent neurologic deficit but with persistent headache symptoms was identified as having cerebral infarction after further evaluation in the outpatient setting. There was one patient each with suspected meningitis due to herpes zoster, third nerve palsy, and vertebral artery dissection, while the remaining patients were diagnosed with a primary headache. The majority of patients presenting to the ED with headaches had primary headaches, accounting for 98% of all headaches [24]. In this study, only 0.46% (2/435) of the patients who underwent brain CT for COVID-19 vaccination-induced symptoms had secondary headaches, and only 1.45% (4/276) of the patients presenting with a headache as their primary complaint had secondary headaches. We expected the number of CT abnormalities to be significantly higher in patients who underwent brain CT after vaccination. However, in this study, none of the patients who visited the ED with headaches after vaccination experienced any other vaccine-related side effects, as confirmed by brain CT.

There are differing opinions in several studies on the number of days after vaccination when post-vaccine side effects become apparent. According to Menni et al., up to 8 days after vaccination was considered the period of occurrence of the side effects of vaccination, and some studies have reported periods ranging from 8 days to more than 2 months after vaccination [9,13,22,25]. We surveyed all post-vaccination headache patients during the study period, and 56% of patients visited the ED with symptoms within 8 days of inoculation; some patients had visited 100 days after vaccination.

In case another pandemic occurs in the future, novel vaccines will again be developed, and it is expected that headaches will still occur after vaccination. ED overcrowding has long been a major issue, and many studies have investigated the causes and improvement strategies [26,27]. The increasing number of COVID-19 or COVID-19 post-vaccination patients visiting the ED has worsened overcrowding in the ED during the COVID-19 pandemic [28,29,30] As per the results of this study, non-enhanced brain CT does not need to be performed if there are no obvious neurological abnormalities in patients who visit the ED after vaccination.

The limitations of this study are as follows. First, this study was conducted at a single institution, and the patients who visited this center were not representative of the patient population of the entire country. Small sample size might fail to adequately represent the broader population, thus constraining the scope for generalization. Furthermore, it is crucial to maintain a balanced representation of different vaccine types for ensuring valid comparisons. An imbalanced distribution could potentially introduce bias, thereby compromising the study’s reliability. Regrettably, the Korean study faced difficulties in procuring data from an equal and substantial number of patients owing to their varied vaccine preferences. Despite striving to examine all patient data from hospital visits, the limited sample size necessitated the amalgamation of the available data for producing results. The results would be more meaningful if there were no patients with abnormalities on the brain CT, and a larger sample could help clearly determine the characteristics of patients with abnormalities on brain CT. Second, as a retrospective study, the effects of confounding factors, such as various underlying diseases and socioeconomic status, cannot be excluded. Indeed, enhancing the granularity of patient categorization could potentially elevate the rigor of this study. Regrettably, during the peak of the coronavirus pandemic, the overwhelming influx of patients hindered our capacity to meticulously document origin, activity level, occupation, and other pertinent demographic determinants within the constraints of the Korean medical infrastructure. In forthcoming research, we aim to address and integrate such determinants as socioeconomic status, occupations, level of activity, type of insurances, and presence or absence of a cohabitant, etc. Third, to assess the relationship between normal brain CT findings and patient prognosis, the patient would need to be followed, especially after 30 or 60 days. Therefore, future studies should consider incorporating follow-up assessments to investigate the relationship between brain CT findings, symptom improvement, and long-term prognosis of patients who present with headaches after COVID-19 vaccinations. Fourth, analyzing data from the first year of vaccination could have had a significant impact on people’s perception and fear of side effects, potentially influencing their decision to visit the ED for post-vaccination symptoms such as headaches. In future retrospective studies, all patients in Korea using the National Emergency Department Information System (NEDIS) or patients who visited multiple institutions or tertiary emergency centers nationwide should be included. By expanding the scope of the study to include patients from multiple institutions or tertiary emergency centers across the country, we can obtain a more representative sample and improve the generalizability of the findings; this approach would allow for a broader analysis of the characteristics, clinical presentations, and outcomes of patients presenting with post-vaccination headaches, considering the potential variations across different healthcare settings. A comparative study using propensity score matching can minimize the effect of confounding factors. By using propensity score matching, the brain CT rates between patients with headaches and a control group during the same period can be compared. This approach helps establish a more rigorous comparison and provides a stronger basis for evaluating the clinical relevance and impact of brain CT in this context. Implementing these suggestions in future studies can enhance the robustness and applicability of the findings and provide more comprehensive insights into the utility of brain CT in patients presenting with post-vaccination headaches. It would also contribute to a better understanding of the healthcare utilization patterns and outcomes associated with this specific population.

In clinical settings, when patients present with headaches, brain CT scans are commonly employed as diagnostic instruments to pinpoint and differentiate the various potential causes of the headache. Nonetheless, the routine or indiscriminate use of brain CT scans can prolong a patient’s duration in the emergency room and may lead to unwarranted medical expenses. This study is the first study to show that non-enhanced CT is not effective for patients who visit the emergency department complaining of headaches after a COVID-19 vaccination. This study serves as a guideline, providing insights for clinicians and headache patients in evaluating the necessity of a brain CT scan, especially in the context of recent coronavirus vaccination. Although it was difficult to reveal statistical significance at one institution and with a small sample size, a chart review was conducted for all patients who fit the inclusion criteria. Based on this study, we hope to draw generalized conclusions about the effectiveness of non-enhanced brain CT after post-vaccination HA by studying all patients in Korea through NEDIS data.

## 5. Conclusions

In conclusion, in this study, conducted at one institution for a year after the start of the vaccination program, brain CT abnormalities were not found in the majority of the patients who visited the ED simply for headaches after a COVID-19 vaccination. However, the type of coronavirus vaccination affects the decision to undergo a brain CT test, and this can be used a tool for differential diagnosis for evaluating headaches in patients who visit the ED. Based on this study, non-enhanced brain CT should be performed cautiously in patients who visit the ED only for headache.

## Figures and Tables

**Table 1:** Demographics of participants during study period.

	Brain CT	No CT	*p*-Value
	N = 435	N = 479	
Chief complaint of headache (%)	246 (56.6)	30 (6.3)	0.000
Age, mean (SD *)	45.74 ± 15.48	42.98 ± 16.58	0.010
Age group, years			
18–29	68(15.6)	123(25.7)	
30–39	103(23.7)	116(24.2)	
40–49	96(22.1)	76(15.9)	
50–59	68 (15.6)	67 (14.0)	
60–69	68 (15.6)	66 (13.8)	
70–79	26 (6.0)	25 (5.2)	
≥80	6 (1.4)	6 (1.3)	
Sex, male, n (%)	144 (33.1)	161 (33.6)	0.871 97
Type of vaccine (%)			0.004
Oxford/AstraZeneca	110 (25.3)	87 (18.2)	
Pfizer-BioNTech	252 (57.9)	323 (67.4)	
Moderna	31 (7.1)	40 (8.4)	
Johnson & Johnson Janssen	30 (6.9)	15 (3.1)	
Unknown	12 (2.8)	14 (2.9)	
Post-vaccination period (mean days)	11.59	10.58	0.294 315
Post-vaccination period <8 days	223 (51.3)	284 (59.3)	0.015
Headache NRS ^†^	2.89	1.44	0.000
Pre-ER visit analgesics (%)	146 (33.56)	129 (26.93)	0.396
Fever (%)	48 (12)	100 (22.9)	0.000
Abnormal CT finding (%)	4 (0.91)		
Brain MR (%)	30 (6.90)	6 (1.25)	0.000
Abnormal MR finding (%)	3 (0.69)	0 (0)	
Disposition (%)			0.000
Discharge	405 (93.10)	413 (86.22)	
Hospitalized	19 (4.37)	28 (5.85)	
DAMA ^‡^	11 (2.53)	35 (7.31)	
Transferred	0	3 (0.63)	
Length of stay	3h32m	3h35m	0.794

* standard deviation. ^†^ Numeric Rating Scale. ^‡^ Discharge against medical advice.

**Table 2:** Cases of abnormal findings on brain computed tomography.

Diagnosis	Age/Sex	C.C	Vaccine Type	Dose	Days after Last Vaccination (Day)	Disposition
Cerebral infarction, ICA dissection *	61/M	HA	AZ	4	18	hospitalized
Cerebral infarction	66/M	Side weakness	AZ	1	30	hospitalized
Cerebral infarction, MCA occlusion	31/M	Side weakness	Pfizer	2	1	hospitalized
Glioblastoma	52/F	HA	Pfizer	1	35	discharge
Rathke’s pouch cyst	19/F	HA	Pfizer	2	32	hospitalized

* through outpatient setting brain CT angiography, ICA, internal carotid artery; MCA, middle cerebral artery.

## Data Availability

Data are contained within the article.
